# Impact of doula's continuous support on serotonin release in parturients: a pilot randomized clinical trial

**DOI:** 10.61622/rbgo/2024rbgo27

**Published:** 2024-04-09

**Authors:** Eleonora de Deus Vieira de Moraes, Mayara Segundo Ribeiro, Cíntia Erbert, Caio Antonio de Campos Prado, Elaine Christine Dantas Moisés

**Affiliations:** 1 Universidade de São Paulo Ribeirão Preto Medical School São Paulo SP Brazil Ribeirão Preto Medical School, Universidade de São Paulo, São Paulo, SP, Brazil.; 2 Women's Health Reference Center of Ribeirão Preto São Paulo st Brazil Women's Health Reference Center of Ribeirão Preto, São Paulo, st, Brazil.

**Keywords:** Doulas, Serotonin, Humanized delivery, Labor, Pregnant women

## Abstract

**Objective:**

To evaluate whether the continuous support provided by doulas influences the endogenous release of serotonin in parturients.

**Methods:**

This pilot study included 24 primigravidae at term. Of these, 12 women received continuous doula support (Experimental Group), whereas the other 12 received the usual assistance without doula support (Control Group). Blood samples were collected from all the women at the active and expulsion stages of labor and at the fourth period of labor (Greenberg period) for evaluation of their serotonin levels using high-performance liquid chromatography.

**Results:**

The average serotonin concentrations in the control and experimental groups were respectively 159.33 and 150.02 ng/mL at the active stage, 179.13 and 162.65 ng/mL at the expulsion stage, and 198.94 and 221.21 ng/mL at the Greenberg period. There were no statistically significant differences in serotonin concentrations between the two groups at the active and expulsion stages of labor. By contrast, within the experimental group, a significant increase in serotonin concentration was observed in the Greenberg period compared with the levels in the active and expulsion stages (p < 0.05).

**Conclusion:**

The novelty of this study relies on the ability to correlate the influence of the continuous support offered by doulas with the release of serotonin in parturients, with the results suggesting that the assistance received during labor can modulate the levels of hormone release in the Greenberg period.

**Brazilian Registry of Clinical Trials:**

RBR-4zjjm4h

## Introduction

In the context of humanized care in childbirth assistance, the use of non-pharmacological resources for both pain relief and the correction of functional dystocia helps toward reinforcing the autonomy of parturients, allowing their active participation during labor and childbirth, as such measures are associated with few contraindications or side effects.^(1-3)^ The continuous support provided by another person appears to be an important resource and is associated with better clinical outcomes during delivery. Women receiving such continuous support are more likely to have spontaneous vaginal deliveries and report a higher satisfaction with the birth experience. Lower rates of pharmacological analgesia, reduced labor duration, and higher Apgar scores in newborns are also observed.^(3)^

Continuous support can be offered by individuals with different competencies, such as health professionals or the spouse, family members, or friends of the parturient. It can also be offered by doulas, who are specially trained companions that provide support during childbirth. However, evidence reveals that better results are obtained when this role is performed by someone external to the hospital or the social support network of the parturient, reinforcing the importance of receiving doula support.^(1,3)^

The importance of doulas has been recognized and recommended by the World Health Organization, and the Ministry of Health of Brazil has supported the incorporation of their practice into the health care team. However, despite scientific evidence highlighting the clear clinical benefits of the support they offer during the labor and post-partum periods, improving perinatal outcomes, doulas still face resistance to their practice in some hospitals in Brazil and little is known about the physiological mechanisms that lead to such results.^(4-7)^ Those are probably influenced by different stimuli that lead to hormone release and activity.

It is known that hormonal activity, particularly that of serotonin, is directly involved in the physiological and emotional changes that occur during the gestational and post-partum periods.^(8-10)^ Studies have shown that pregnant women present increased concentrations of serotonin in their cerebrospinal fluid and plasma during the gestational and post-partum periods compared with the general levels in non-pregnant women.^(10,11)^ However, data on the physiology of the serotoninergic system during labor and in the immediate postpartum period remain scarce.

Serotonin, a monoamine molecule also known as 5-hydroxytryptamine (5-HT), acts systemically as a hormone and centrally as a neurotransmitter. Its hormonal activity includes a wide range of physiological functions. Centrally, serotonin acts as a synaptic inhibitor, suppressing synapses involved in pain sensation and modulating mood and motivation.^(12)^ During labor, it regulates uterine contractions by binding to 5-HT2A receptors. Additionally, it induces the expression of uterine collagenase after delivery, thereby promoting uterine involution in the Greenberg period.^(13-15)^ Recent studies have also shown that serotonin regulates the initiation and maintenance of maternal behavior in rats, such as in care interactions with their pups.^(16,17)^

This present study was carried out to determine whether the continuous support offered by doulas influences the release of serotonin in parturients. As the first study of its kind, we sought to investigate the physiological bases for the applicability of this mild technological resource, with the additional aims of improving our understanding and optimization of the safe practices of humanized obstetric care.

## Methods

This pilot study, randomized, open-label, controlled clinical trial was developed at the Reference Center of Women's Health of Ribeirão Preto - MATER (Centro de Referência da Saúde da Mulher de Ribeirão Preto - CRSMRP-MATER) in S*ão* Paulo, Brazil. The study was approved by the Research Ethics Committee of the Clinical Hospital of the Faculty of Medicine of Ribeirão Preto of the University of São Paulo (*Hospital das Clínicas da Faculdade de Medicina de Ribeirão Preto da Universidade de São Paulo -* HCFMRP-USP) (Reference No. 2.140.965, CAAE No. 69651917.2.0000.5440) as well as by the Research Committee of CRSMRP-MATER. The study was duly registered as a randomized controlled trial on the Brazilian Registry of Clinical Trial (*Registro Brasileiro de Ensaios Clínicos* - ReBEC) platform (Code No. RBR-4zjjm4h) and is part of a larger research project, originally titled "Randomized Clinical Trial: Influence of Continuous Support Offered by Doula on Release of Oxytocin, Cortisol, Melatonin, Adrenaline, Noradrenaline, Serotonin and β-endorphin in Parturients".

The data, collected between June 2018 and June 2020, included information obtained at term from primigravidae with a single gestation. Parturients were admitted to the institution at the beginning of the active stage of labor to receive birthing assistance. The inclusion criteria was primigravidae with a single gestation at term, without comorbidities and admitted to the institution at the beginning of the active stage of labor.

The following were the study exclusion criteria: parturients with no companion of choice (family members, friends, spouse), late gestational loss (fetal death), and comorbidities that could interfere with the endogenous release of serotonin, like pancreatic diseases and enteropaties. The discontinuation criteria were pregnant women who decided to abandon the study, pregnant women who needed to receive drugs known to interfere with hormone parameters, and labor resolution through urgent/emergency obstetric procedures.

Since a study of this type has never been published in the literature, it was developed as a pilot project. Birkett (1994)^(18)^ recommended the inclusion of at least 10 patients per group to obtain a good estimate of the variance, which then can be used to calculate the cohort size of future clinical trials. A randomization scheme was implemented using the R program package "blockrand", which generated 13 blocks of 8, 4, 4, 4, and 2 sizes. The randomization sequence was shown in a table and strictly observed by the researchers, who followed the sequence and applied it to the women admitted in labor during their shift. The researchers did not previously know which parturients would be in each group. Each study participant was informed of their group assignment after signing an informed consent form.

Using simple comprehensible explanations, all pregnant women were clearly and objectively informed about the aims of the project and the practiced research protocol. The informed consent form was signed either by the pregnant woman participating in the study or by the legal guardian in the case of adolescent participants under 18 years of age.

Parturients were free to refuse to participate in the study or withdraw their consent to participate at any stage. It is worth emphasizing that in adherence to institutional care protocols, this study did not interfere with the obstetric care provided to the selected participants.

The 24 parturients included in the study were randomly distributed into either the Experimental Group (EG) or the Control Group (CG). Those in the EG received the continuous support offered by doulas in addition to the usual obstetric assistance. The women in the CG received only the usual assistance, that consists in care routines for usual risk birth. All women in both groups chose their companion themselves.

The continuous doula support was offered as a 1:1 care model at the time of admission and lasted from the active labor stage to the Greenberg period (fourth period of labor). The assistants were a group of five women who were duly certified as doulas after having been trained and registered as members of the project research team. The intervention consisted of one of those doulas offering continuous support to one parturient at a time in three different aspects: physical comfort and emotional and informational support.

For physical comfort, the following measures were offered: a massage in the lumbosacral region as well as in the upper and lower limbs, performed using slip, kneading, and pressure techniques along with the use of a vegetable massage oil certified by the National Health Surveillance Agency (Agência Nacional de Vigilância Sanitária (ANVISA)); hydrotherapy in a shower bath, at a temperature controlled to the parturient threshold (between 34°C and 38°C); warm water compresses (between 38°C and 40°C), applied to the lumbosacral and hypogastric regions; use of a Swiss ball to favor the possibility of vertical rest (with the parturient seated with the trunk leaning forward, with pillow support on the stretcher), pelvic mobility with anteversion, and pelvic retroversion and circumference; comforting touches; and ambulation and changes of posture, according to the preference of the parturients, who may adopt left lateral decubitus, four-support, sitting, standing, kneeling, or squatting positions during contractions.

For emotional support, a continuous empathic and welcoming presence was offered, with the doula providing encouragement and security and seeking to assist the parturient in identifying her emotions and bodily sensations. The informational support involved offering information about the evolution of labor, ensuring the normality of the process, replying to main doubts, and favoring communication between the parturient and the team.

According to the methodologies described by Kennell et al. (1991),^(19)^ Gallo (2015),^(20)^ and Hodnett and Osborn (1989),^(21)^ the continuous support offered by the doula was personalized, seeking to meet the needs of each parturient in the different stages of their labor using the resources described above to promote their comfort and autonomy. As a measure to ensure the consistency and reliability of the experimental intervention, the methods provided to each parturient were documented on a record sheet, and periodic research team meetings were conducted, thus verifying the homogeneity of the interventions offered.

A peripheral venous catheter was placed in all parturients, with a No. 20 scalpel used to gain venous access (when collecting blood for the laboratory tests needed to provide obstetric care). Venoclysis was maintained by infusion with 3 mL of 0.9% saline solution, and blood samples were collected during the active (6–9 cm of dilation) and expulsion stages (10 cm of dilation) and immediately after birth (Greenberg period). This allowed for the plasma serotonin concentrations to be plotted as a function of the evaluated periods. At each predetermined blood collection time, approximately 3 mL of blood was first withdrawn with a syringe and discarded to avoid interference with the dosages to be performed. Subsequently, 10 mL of blood was collected into a non-heparinized syringe and then transferred to EDTA-containing tubes. After each blood collection, the venous catheter was infused with 3 mL of saline solution. The blood samples were processed in tubes containing a clot activator and immediately centrifuged at ambient temperature at 3,400 rpm for 10 min. The resultant plasma was aliquoted and sent to the laboratory for analysis. The serotonin concentrations were determined and analyzed using high-performance liquid chromatography according to the methods described by Kremer et al. (1990)^(22)^ and Mohri et al. (2005).^(23)^

For data analysis, the following variables were considered: (i) demographic: age, ethnicity, education, marital status, education level, and professional activity; (ii) clinical data: anthropometric and nutritional status (weight, height, and body mass index); (iii) maternal clinical outcomes: duration of active labor stage, duration of expulsion stage, and need for pharmacological analgesia, oxytocin, episiotomy, forceps, or vacuum extractor; (iv) neonatal clinical outcomes: Apgar score and anthropometric measurements of newborns; (v) serotonin concentration.

Initially, an exploratory analysis of the data was performed using measures of central position and dispersion. Qualitative variables were summarized with consideration of the absolute and relative frequencies. Comparisons between the groups in relation to the sociodemographic variables were performed by applying the chi-square test for the qualitative variables, whereas the non-parametric Wilcoxon test for independent samples was applied for the quantitative variables.

A mixed-effects model for linear regression was built to compare the serotonin concentrations between the two groups at each labor stage and between the three stages within each group. These comparisons were performed with consideration of orthogonal contrasts. Analysis of residuals was conducted to verify if the model fits the data. The model was implemented in the SAS version 9.4 program.

## Results

**Figure 1:**
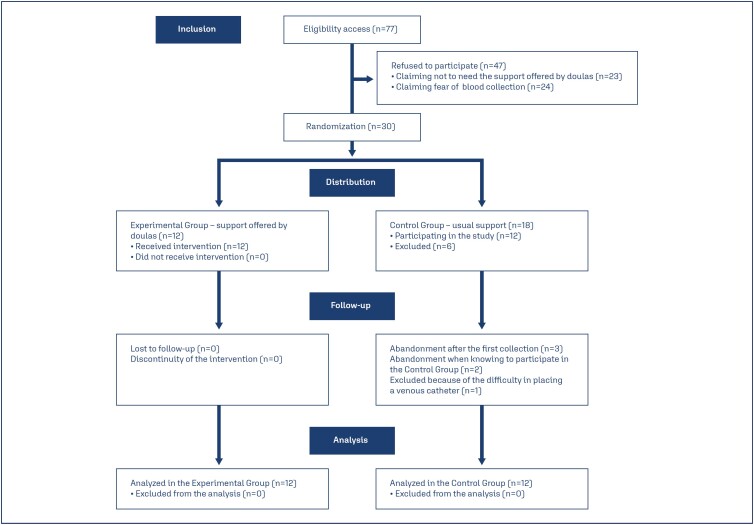
CONSORT flow diagram applied to the study "Impact of doula's continuous support on serotonin release in parturients: a pilot randomized clinical trial"

In total, 77 parturients were considered eligible for the study. Of these, 30 agreed to participate and were randomly distributed between the two groups. There were no exclusions at this stage of the study. However, six parturients in the CG abandoned the study; of these, three left right after collection of the first biological sample, two left after finding out they would be in the CG, and one left because of difficulties in placing the venous catheter. Therefore, 24 healthy women (aged between 15 and 38 years) were finally included in the study, with 12 in the EG and 12 in the CG (Figure 1).

The data of the continuous and categorical maternal sociodemographic and clinical variables are presented in table 1. The groups appeared homogenous in terms of these data. There was no significant difference between the groups regarding age, years of study, gestational age, body mass index, prenatal care appointments, marital status and ethnicity. Regarding marital status, in both groups the participants declared themselves single, being accompanied at the time of birth by their partner, mother or mother-in-law. Only one parturient from the CG and two from the EG attended the pregnancy course during pregnancy, showing the homogeneity of the samples.

**Table 1:** Continuous and categorical variables characterizing the 24 parturients in the study

Variables, mean (standard deviation)		Experimental Group n(%)	Control Group n(%)	p-value
Age (years)||		23.3(7.97)	21.33(5.05)	0.70‡
Years of study (years)||		10.33(2.71)	10.67(1.92)	0.62‡
GA* (weeks)||		38.92(1.31)	39.00(1.21)	0.92‡
BMI† (kg/m^2^)||		30.86(4.91)	28.17(5.51)	0.14‡
Prenatal care appointments||		9.08(2.61)	9,17(2.12)	0.88‡
Marital status¶	Married	5(41.67)	7(58.33)	0.69§
	Single	7(58.33)	5(41.67)	
Ethnicity¶	White	8(66.67)	5(41.67)	0.22§
	Black	7(58.33)	4(33.33)	
Companion at birth¶	Partner	7(58.33)	6(50.00)	0.91§
	Mother	4(33.33)	5(41.67)	
	Mother-in-law	1(8.33)	1(8.33)	
Course for pregnant women¶	Yes	2(16.67)	1(8.33)	0.53§
	No	10(83.33)	11(91.67)	

* GA - gestational age;

† BMI - body mass index

‡ *p*-value according to the Wilcoxon–Mann–Whitney test for independent samples

§ *p*-value according to the chi-square test

|| Mean (standard deviation)

¶ Number (percentage)

No significant differences were found regarding maternal and neonatal obstetric outcomes, as shown in table 2. All participants in the CG had vaginal births, while two participants in the EG had cesarean section (18.18%). No patient underwent the episiotomy procedure in both groups and the laceration rate in both groups was the same, with two parturients in each group (18.18%). The use of misoprostol for labor induce was 18.18% in the EG (two parturients) and 9.09% in the CG (one parturient). Four participants in each group received synthetic oxytocin (36.36%) for induction or correction of functional labor dystocia. Seven parturients in the EG (63.64%) and six parturients in the CG (54.55%) received combined pharmacological analgesia. No participant in the two groups delivered using forceps and one woman in the control group delivered using a vacuum extractor. Regarding the estimated duration of labor, the EG had an average of 8.27 hours (SD = ± 2.10) and 6.64 hours (SD = ± 2.66) in the CG. As for the duration of expulsive, the EG had an average of 0.96 hours (SD = ± 0.27) and the CG had an average of 0.73 hours (SD = ± 0.29).

**Table 2:** Maternal and neonatal obstetric outcomes for the 24 parturients in the study

Variables		Experimental Group n(%)	Control Group n(%)	p-value
Delivery route*	Vaginal	10(83.33)	12(100)	0.14†
	Cesarean	2(16.67)	0(0)	
Misoprostol*	Yes	2(16.67)	2(16.67)	1.00†
	No	10(83.33)	10(83.33)	
Oxytocin*	Yes	5(41.67)	4(33.33)	0.67†
	No	7(58.33)	8(66.67)	
Analgesia*	Yes	8(66.67)	7(58.33)	0.67†
	No	4(33.33)	5(41.67)	
Laceration* §	Yes	8(80.00)	10(83.33)	0.84†
	No	2(20.00)	2(16.67)	
Episiotomy* §	Yes	0(0.00)	1 8.33)	0.35†
	No	10(100.00)	11(91.67)	
Labor duration||		8.27(2.10)	6.64(2.66)	0.12‡
Expulsion duration||		0.81(0.44)	0.75(0.29)	0.49‡
Apgar 1st minute||		8.00(1.91)	7.83(1.27)	0.52‡
Apgar 5th minute||		9.33(0.98)	9.25(0.62)	0.47‡
Newborn weight (g)||		3,049.33(452.81)	3,235.92(422.74)	0.37‡

* Number (percentage);

† p-value according to the chi-square test;

‡ p-value according to the Wilcoxon–Mann–Whitney test for independent samples;

§ For vaginal deliveries

|| Mean (standard deviation)

**Figure 2:**
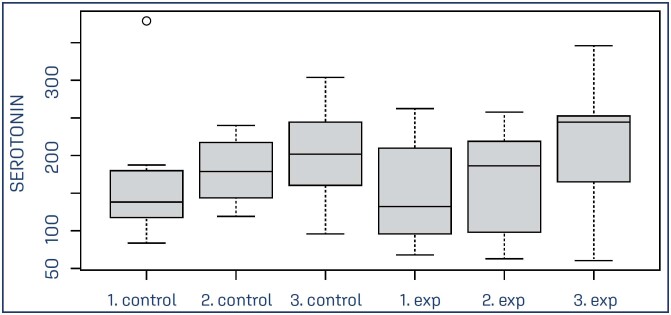
Boxplot of endogenous serotonin concentrations (ng/mL) according to clinical stages of labor in the control group and in the group of parturients receiving continuous doula support

For all participating parturients in the study, it was possible to collect biological blood samples at three different times: active phase (T1) - varying between 6 and 9 cm of dilation, expulsive phase (T2) and in the fourth Greenberg period, immediately after birth (T3). Data analysis using the mixed-effects linear regression model showed no significant difference between the groups, when evaluating serotonin concentrations in the respective stages of labor between the EG and CG (T1: p = 0.7; T2: p = 0.52; T3: p = 0.44) (Table 3) (Figure 2).

**Table 3:** Plasma serotonin concentrations according to clinical stages of labor in the 24 parturients in the study

Blood collection stages	Experimental Group (ng/mL)	Control Group (ng/mL)	p-value*
Active stage (T1)	150.02 (66.57)†	159.33 (76.72)†	0.7102
Expulsion stage (T2)	162.65 (67.30)†	179.13 (42.17)†	0.5115
Greenberg period (T3)	221.21 (84.52)†	198.94 (58.88)†	0.3757

* p-value according to the Wilcoxon–Mann–Whitney test for independent samples;

† Mean (standard deviation)

Post hoc analyzes showed, however, significant differences in intragroup serotonin release in the EG, showing an increase in serotonin concentration between T1 and T3 (p = 0.0032) and T2 and T3 (p = 0.0069). There was no significant difference between T1 and T2 (p = 0.77) of the EG. In the control group, there was no statistical difference in serotonin release between times T1 and T2 (p = 0.51), T1 and T3 (p = 0.14) and T2 or T3 (p = 0.40) (Table 4) (Figure 2).

**Table 4:** Comparisons of plasma serotonin concentrations between and within the control and experimental groups of the 24 parturients in the study

Comparisons	Estimate of difference between means	p-value**
(CG* – EG†)|| T1	9.3167¶	0.7102
(CG* – EG†)|| T2	16.4750¶	0.5115
(CG* – EG†)|| T3	–22.2667¶	0.3757
CG* (T1 – T2)§	–19.7617¶	0.4307
CG* (T1 – T3)§	–39.6083¶	0.1179
CG* (T2 – T3)§	–19.8167¶	0.4301
EG† (T1 – T2)§	–12.6333¶	0.6144
EG† (T1 – T3)§	–71.1917¶	0.0060††
EG† (T2 – T3)§	–58.5583¶	0.0225††

* CG - Control Group;

† EG - Experimental Group;

§ Comparisons between groups at each time (T1, active stage; T2, expulsion stage; T3, Greenberg period);

|| Comparisons between times within each group;

¶ Estimated difference between means;

** p-value according to the Wilcoxon–Mann–Whitney test for independent samples, concentrations expressed in ng/mL;

†† Significant p-value (p<0.05)

## Discussion

The CG and EG were homogeneous with regard to the sociodemographic variables and clinical findings, which did not influence the data related to the serotonin concentration dynamics in the studied perinatal and postnatal periods. Likewise, no significant differences were found between the two groups in terms of maternal and neonatal obstetric outcomes. However, in the EG, the serotonin concentrations observed during the Greenberg period were significantly different from those in the active and expulsion stages, respectively, showing that there was an increase in the endogenous production of serotonin in the group that received the continuous doula support. This difference among the studied periods was not observed in the control group.

To date, there are no published studies showing the levels of serotonin in the blood or cerebrospinal fluid during the Greenberg period. In this context, the present study is the first to show the correlations between the concentration of the hormone, the continuous support, and the clinical stages of labor, confirming the increase in serotonin in the immediate postpartum period.

However, correlations between intrapartum serotonin concentrations and labor pain as well as between serotonin and postpartum depression and uterine involution in the third period of labor have been published in the literature.^(10,12,17,24)^ Thus, it is possible to raise some hypotheses about the unpublished findings of this pilot study from the point of view of the central and systemic activities of serotonin.

From the central activity perspective, studies using animals and cell cultures have provided clues to the possible function of increased systemic serotonin concentrations. It is likely that increased intrapartum and immediate postpartum serotonin concentrations are involved in the regulation of uterine levels through 5-HT2A receptors.^(13,15)^ Furthermore, serotonin has been shown to induce uterine collagenase expression after delivery, promoting the uterine involution required in the Greenberg period.^(13)^

Although not observed in this study, scientific evidence has pointed to an association between continuous support and a greater satisfaction with the labor experience, which may be directly or indirectly related to serotonin-induced mood and behavior regulation functions.^(3)^ Indeed, the support offered to women during childbirth appears to be effective in reducing the adverse consequences of fear and distress associated with this period.^(25)^ Studies using peripheral measures of serotonergic activity have concluded that women with postpartum depression have lower concentrations of available tryptophan, low blood serotonin levels, and altered binding of serotonin to the corresponding transport sites on platelets.^(10,16,17)^

Sekiyama et al. (2013)^(10)^ conducted a study on 28 healthy lactating women to assess the relationship between mood states and blood 5-HT levels during the early and late postpartum periods. That study was the first to report an increase in blood serotonin concentrations accompanied by a reduction in tension/anxiety during the late postpartum period in healthy lactating women, indicating the important role of the serotoninergic system in regulating tension and anxiety in healthy puerperal women.^(10)^

In a 2017 Cochrane systematic review on continuous support, many studies of postpartum depression were analyzed, although they could not be combined owing to the differences in their methodologies.^(3)^ However, two trials found that fewer women developed depressive symptomatology when labor support was offered, although it may have been an incidental outcome in one of the studies (low-quality evidence).^(3)^ Considering that a decrease in serotonin is implicated in postpartum depression in studies described in the literature and the fact that an increase in serotonin was found in the Greenberg period in the doula-assisted parturients of this present study, future investigations should be carried out to evaluate the correlation between serotonin concentrations in the fourth period of labor and the mechanisms related to postpartum depression.

The discovery of an increase in serotonin levels during the Greenberg period in doula-assisted parturients may also be interesting for future studies that investigate the establishment of immediate attachment relationships between mother and newborn. Although there are still no definitive conclusions on how serotonin acts in the direct establishment of attachment relationships, it is known that its increase directly effects the maternal behavior of mammals, such as in the approach and recovery of pups, licking behavior, and more time spent in the nest by the mother.^(16,17)^

Regarding prenatal care, future studies could explore the hypothesis that the involvement of doulas during pregnancy in prenatal meetings that provide emotional support and childbirth preparation may affect hormonal concentrations, obstetric and neonatal outcomes. These same variables could be explored with the doula initiating continuous support earlier, in the latent labor stage.

The novel findings in this study reaffirm the importance of recommending continuous intrapartum support and practices that aim to incorporate doulas as an integral part of childbirth care. The increase in serotonin levels during the Greenberg period in doula-assisted parturients, as seen in this study, is interesting and warrants future research with a larger sample size, which could further elucidate the physiological effects induced by continuous support and the neuroendocrinology of parturition.

Although the sample size is adequate for a pilot project, an expanded casuistic is recommended for better extrapolation of the hormonal dosages results. The fact that parturients lacked knowledge about both the role of doulas and the importance of having continuous specially trained support was observed as a factor hindering their acceptance to participate in the study. Enabling doulas to participate in prenatal courses where the study is being conducted could facilitate their acceptance in future investigations.

## Conclusion

This pilot study yielded original research results that can be used as a methodological basis for future investigations on the subject. With the present cohort, it was verified that the continuous support offered by doulas did not influence the endogenous release of serotonin in parturients. However, the intervention induced an increase in the release of the hormone during the Greenberg period compared with the levels observed in the previous clinical stages of labor, suggesting that the presence of a doula during labor might influence the levels of serotonin release during the fourth period of labor. The hormonal repercussion might explain, at least in part, the literature-documented benefits of the support offered by doulas.
